# Immunomodulatory effects of aflatoxin B1 (AFB1) and the use of natural products to ameliorate its immunotoxic effects: A review

**DOI:** 10.12688/openresafrica.14406.2

**Published:** 2025-02-07

**Authors:** Gilbert Kipkoech, Mercy Jepkorir, Sally Kamau, Alice Wanyoko, Susan Kibunja, Rechard Amozi Jeremiah, Johnson Masese, Vincent Ntui-Njock, Charles Mutai, Peter Mwitari

**Affiliations:** 1 Kenya Medical Research Institute, Nairobi, Kenya; 2 Kenyatta University, Nairobi, Nairobi County, Kenya; 3 Pan African University Institute for Basic Sciences Technology and Innovation (PAUSTI), Nairobi, Kenya; 4 Masinde Muliro University of Science and Technology, Kakamega, Kakamega County, Kenya; 5 H3Africa, Buea, Cameroon

**Keywords:** Aflatoxin B1, mycotoxin, immunotoxic, immunomodulatory, immunosuppression, natural products

## Abstract

Aflatoxin B _1_ (AFB _1_), a potent mycotoxin, is known to be an immunotoxic agent that causes various immune system disorders. It can cause immunosuppression by direct toxic effect on the host or by its ability to interfere with the immune response and thereby lead to secondary infections. The immunomodulatory effects of AFB _1_ and its metabolites have been reported in many studies. Yet, the exact mechanisms responsible for these immunomodulatory effects are still obscure. This review summarizes the current findings on the interaction between AFB _1_ and the host immune system, while also highlighting other potential mechanisms involved in AFB _1_-induced immunosuppression. These potential mechanisms include modulation of cytokine production, production of inflammatory mediators and their receptors, activation of pro-inflammatory pathways, alteration in cell surface receptors involved in T cell activation and regulation of apoptosis. The review also discusses the findings on natural products that have been found capable of inhibiting AFB _1_-induced immunosuppression in various animal models. With the latest exploration of natural products as a solution, the burden of aflatoxicosis in society is likely to subdue. Some of the promising natural products that have been highlighted to have ameliorative effects include grape seed proanthocyanidin extract (GSPE), bentonite clay, smectites clay and *Spirulina plantesis* among others. Considering the seriousness of aflatoxicosis in the public domain and lack of effective management approaches, there is an urgent need for more research to understand AFB _1_-induced immunotoxicity and possible remedies.

## Introduction

Aflatoxin is a secondary metabolite synthesized by a few particular types of mold ^1^. The most common producing strains include *Aspergillus flavus* and *Aspergillus parasiticus.* These strains propagate on food crops such as maize, groundnuts and other oil-producing seeds ^2^. Aflatoxin contamination of food is associated with acute illnesses that may result in death or hepatocellular carcinomas. According to statistics, more than 4 billion people globally are affected by aflatoxin contained in foods ^3^. Today, more than 100 countries in the world have imposed aflatoxin regulations that are meant to safeguard animals and human health. Aflatoxins not only have caused health effects on animals and humans but also economic losses especially in countries that export maize and other susceptible food crops ^4^. The major forms of aflatoxin that occur in various food types include B _1_, B _2_. G _1_ and G _2_. Of all these types, the predominant and most toxic form is aflatoxin B _1_^2^.

The contribution of aflatoxin to pathogenesis of diseases such as liver cancer and immune suppression has been widely studied ^5, 6^. Research findings have documented that aflatoxin B _1_ is a hepatocacinogen in animal models ^7^. Apart from carcinogenic ^1^ and other chronic toxicities, studies have also shown immunosuppressive effects. In a study by Qian *et al*. (2014) ^8^, aflatoxin B _1_ was shown to reduce the expression of interleukin 4 (IL-4) and upregulate the expression of proinflammatory cytokines inteferon-γ (INFγ) and tumor necrosis alpha (TNFα) in rats. Aflatoxin B _1_ has been shown to suppress immunity by decreasing protective effects of vaccines ^9^. Immunotoxic effects of aflatoxin B _1_ are mainly felt by cell-mediated immunity, where it is able to decrease the levels of activated lymphocytes, suppress lymphoblastogenesis and impair delayed-type hypersensitivity ^10^. In human subjects, aflatoxin B _1_ is capable of changing immunological parameters in both cell-mediated and humoral immunity. High doses of aflatoxin B _1_ have a greater impact on cellular immunity thereby resulting in decreased host resistance to pathogenic microbes ^11– 13^. Studies have also shown the impact of AFB _1_ ingestion on the levels of immunoglobulins. Ingestion of aflatoxin B _1_ by birds leads to decreased levels of immunoglobulins IgM, IgA and IgG ^14^.

## Aflatoxin B _1_ (AFB _1_): Sources, occurrence, and exposure

Aflatoxin B _1_ is mostly found in food products, like corn, cotton, peanuts and tree nuts. The presence of AFB _1_ in these crops is influenced by several factors including temperature, humidity and insect damage ^15^. Moreover, AFB _1_ can also be present in animal feed since it can be produced by mold that grow on feed ingredients. The biosynthesis of aflatoxin B _1_ is controlled by factors related to stress, like reactive oxygen species (ROS) and enzymes such as superoxide dismutase (SOD) and catalase (CAT) ^16^. These oxidative stress elements play a role in both sclerotial differentiation (SD) as well as the production of aflatoxin B _1_ in *A. flavus*. The peroxisomes and aflatoxisomes, which are organelles involved in responding to stress ^16^, are responsible for the production of aflatoxins. The production process of AFB _1_ is regulated by a combination of stress factors including lipid hydroperoxide, superoxide, hydroxyl radicals and thiyl radicals ^16^. The link between stress and both SD and the biosynthesis of aflatoxin B _1_ indicates that oxidative stress plays a role in the contamination of agricultural produces with AFB _1_.

When it comes to exposure to AFB _1_, it mainly occurs through consuming foods, especially cereal crops, such as corn and peanuts. AFB _1_ has the potential to enter the food chain at various points such as before harvesting, after harvesting and during processing and storage ^17^. When people consume food contaminated with AFB _1_, the toxin can be absorbed into the body system and then spread to different organs in our body. The liver is particularly vulnerable to its effects ^18^.

## Methodology

A comprehensive literature review was carried out during the study. All the articles related to the topic were reviewed and evaluated. A thorough review and structured approach was used when selecting information from the credible sources. Scientific databases -Google Scholar, Scopus and PubMed -were used to obtain credible sources related to the study. To get these sources, search terms "Aflatoxin B _1_," "immunomodulation," "immune system," "natural products," "phytochemicals," "immunotoxicity," were used. Age limit was not used to filter the articles. Publications were selected based on their relevance and scientific rigor. All the articles reporting on immunomodulatory effects of aflatoxin B _1_ and or natural products with respect to immune function were selected. Conference abstracts, editorials, and opinion articles were excluded. The data collected from the articles are authors names, year of publication, journal details, name of natural products used to ameliorate immunotoxic effects of AFB _1_ and main outcomes related to Aflatoxin B _1_ immunomodulation and natural product interventions.

## Immunotoxic mechanism of Aflatoxin B _1_

**Figure 1.**
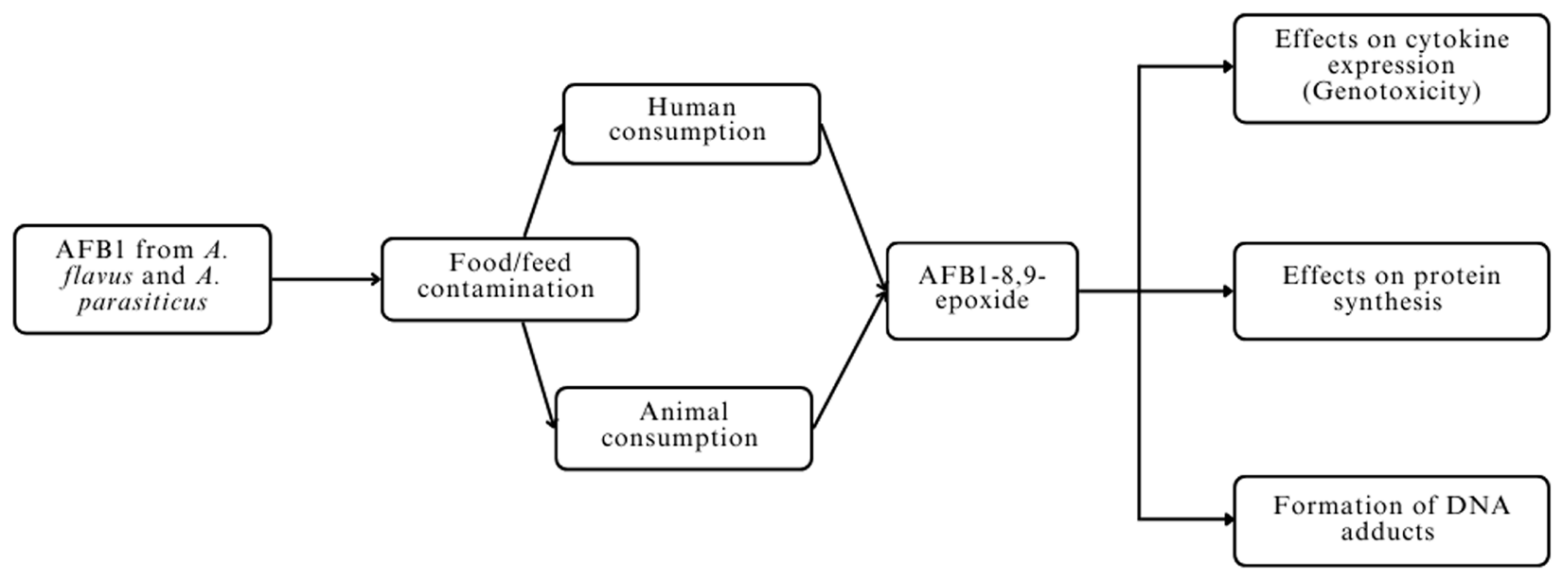
Immunotoxic mechanism of aflatoxin B1.

AFB _1_ has been shown to affect the immune systems at various levels. The mechanism of action of AFB _1_ on the immune system is widespread and has not been clearly elucidated. From previous studies, aflatoxin B _1_ or its activated metabolites exhibit an interaction with cellular proteins, including cytochrome enzymes ^19, 20^. This kind of interaction impedes basic metabolism and synthesis of proteins. This eventually result in cell death ^21^. Aflatoxins are hepatoxic and genotoxic in humans and susceptible animals. Upon consumption, aflatoxin is broken down in the liver by cytochrome P450 (CYP450) enzymes. Notably, CYP1A2 and CYP3A4 are responsible for metabolism of aflatoxins ^22^. In the liver, aflatoxin is metabolized to active metabolite aflatoxin-8,9-epoxide which is a ROS. The production of ROS is exacerbated in the presence of AFB _1_. A study by Ma *et al.* (2021) ^23^ showed that the intracellular levels of ROS increases significantly in the presence of AFB _1_. The resulting ROS is capable of binding DNA, thus damaging it ^22^. In addition, the active metabolites may bind to proteins, thus rendering them non-functional. According to Guengerich *et al*., (1998) ^24^, CYP450 isoenzymes, CYP1A2 and CYP4A4, play a critical role in the metabolism of AFB _1_ to generate a highly reactive compound, *exo-*8,9-epoxide. The formation of this highly reactive compound is an important step in the genotoxic pathway of aflatoxin. *Exo-8,*9-epoxide has the capacity to alter the conformation of DNA ^25^ and binds strongly to guanine bases in DNA to form aflatoxin-N7-guanine. According to Raney *et al*. (1993), the epoxide formed interferes with the role played by DNA-dependent RNA polymerase and consequently lead to a reduction in synthesis of RNA and proteins. The metabolism of aflatoxin into highly active metabolites may directly or indirectly interfere with proliferation and differentiation of immune cells and production of cytokines ^26^. The immunotoxic mechanism of aflatoxin B _1_ is shown in Figure 1.

The metabolism of AFB _1_ yields genotoxic metabolites, mainly 8,9-epoxide-AFB _1_, which binds to DNA to form aflatoxin B _1_-DNA adducts ^24^. The adducts formed have the capacity to interfere with normal processes, including replication and transcription. This eventually leads to formation of DNA lesions in the dsDNA. If the exposure to AFB _1_ is prolonged, more lesions are formed in the DNA and this will result in mutation and carcinogenesis ^27^. Apart from the genotoxic effects of AFB _1_, continuous exposure has been linked with severe consequences such as growth retardation, suppression of the immune system and teratogenic effects in human and farm animals. Yet, the exact mechanism of aflatoxin B _1_ has not been fully understood ^28^. Epigenetic modifications are inherited variations in how genes operate or expression of phenotype which have no impact on the nucleotide base pairings of the DNA strand ^29^. Hepatocellular carcinoma may develop as a result of epigenetic changes brought about by AFB _1_ exposure, including DNA methylation, non-coding RNA, and histone alteration, which may change the expression of certain genes right away or later.

## Immunomodulatory effects of aflatoxin B _1_

### Effects on the components of innate immunity

Innate immunity is a first line of defense that consists of cells and mechanisms responsible for elimination of foreign antigens in the body ^30– 32^. The major components of innate immunity are physical epithelial barriers, phagocytic leucocytes, complement system, dendritic cells and natural killer cells ^33, 34^. The skin is a major organ that prevents entry of foreign particles into the body. There is evidence that AFB _1_ has tumorigenic effects on the skin. For example, a study by Rastogi *et al*. (2006) ^35^ found that topical application of aflatoxin B _1_ leads to the development of tumors. Aflatoxin B _1_ can easily penetrate human skin and cause toxic effects. In animal models treated with aflatoxin B _1_, some of the evident clinical symptoms included hair loss in the skin ^36^. Epithelial cells are found in most soft tissues and form a physical barrier to most pathogens. Effects of aflatoxin in epithelial cells have been widely studied ^37, 38^. AFB _1_ and its hydroxylated metabolite, aflatoxin M _1_ (AFM _1_) have been reported to be cytotoxic to many cells including epithelial cells. The AFM _1_ is excreted through the milk, and therefore it is important in aflatoxicosis in young mammals and infants. Wu *et al*. (2021) ^37^ in their study found that aflatoxin B _1_ and its hydroxylated metabolite, AFM _1_, causes inhibition of cell proliferation, apoptosis and arrests cell cycle. Wu *et al*. (2021) also noted that AFB _1_ and AFM _1_ brought about alteration of related gene expressions.

The intestinal innate system has also been shown to be adversely affected by dietary aflatoxin. Gastrointestinal mucosa form a barrier to ingested pathogens and there have been concerns over the damage caused by AFB _1_. In their study, Gaikwad and Pillai (2004) ^39^ sought to study the effects ingested aflatoxin B _1_ on the morphology and histochemical properties of the gastrointestinal tract (GIT) in mice. Their results indicated that there were significant changes in morphology and histochemical properties of GIT among the mice administered aflatoxin B1. AFB _1_ was shown to severely damage the mucosal membranes and significantly decrease glycoprotein levels in the GIT. Gaikwad and Pillai concluded that aflatoxin B1 found in contaminated food may affect glycoprotein synthesis and affect the protection of GIT lining. Wang *et al*. (2018) ^40^ reported that histopathological injuries in GIT, structural changes and decreased expression of TLR particularly in the small intestines after AFB _1_ exposures. Moreover, the histopathological observations indicated that the villi in the ileum of broiler chickens fed with AFB _1_ were shedding. The goblet cells in the intestinal tract of broilers exposed to aflatoxin B _1_ largely came down. At the molecular level, aflatoxin B _1_ is capable of decreasing mRNA expression of toll-like receptors 2, 4 and 7 in the ileum ^40^. 

The immunomodulation effects of AFB _1_ on human natural killer cells have been studied ^41, 42^. Natural killer cells are component of innate immune system that plays a role of eliminating abnormal cells, including virally infected and tumorigenic cells ^43^. It has been shown that aflatoxin B1 exhibits immunosuppressive effects on NK cell activity of healthy humans ^44^. However, a study condcted by Sabourin *et al*. (2006) ^45^ found that splenic T-cells (CD4 ^+^ and CD8 ^+^), B-cells (CD19 ^+^) and NK cells (CD3 ^−^/NK1.1 ^+^) levels remained unchanged after aflatoxin B _1_ aerosol exposure of female mice as evaluated by flow cytometry analysis.

Phagocytosis is a fundamental process in innate immunity ^46^. Phagocytic cells of innate immunity include neutrophils, macrophages, eosinophils and dendritic cells. According to Coulombe (1993) ^47^, aflatoxins have a significant impact on various cell population in the thymus. For instance, their study found that the population of macrophages and their phagocytic activity was reduced under pathological state induced by AFB _1_. Aflatoxins were also reported to reduce the activities of T-cell-dependent roles of mouse splenic lymphocytes. The role of phagocytic cells such as natural killer cells is depressed by aflatoxin B _1_^44, 47^.

The innate immune system serves as the first line barrier against pathogenic attack, and it is notably important during invasion by bacteria or viruses in the mucosal membranes ^48^. A number of studies has noted the effects of aflatoxins on different components of the innate immune system. Aflatoxins have been reported to impair the ability of cells of innate immunity to recognize foreign particles mainly by repressing the transcription of important receptors on the cell surface ^40^. In their study, Wang *et al*. (2018) ^40^ reported that feeding the broilers with aflatoxin B _1_ repressed the transcription of TLR-2, TLR-4 and TLR-7. This implies that aflatoxin B _1_ suppresses the activity of innate immunity through the receptors involved in recognition of pathogenic microorganisms. It is also worth noting that the antigen-presenting activity of some phagocytic cells, for instance dendritic cells, is reduced as a result of aflatoxin B _1_, although this reduction could not be linked to repression of toll-like receptors or specific cytokines ^49^.

The complement system is an important arm of the innate immune system. This system is mainly responsible for activating phagocytosis of pathogenic microbes. Studies have shown that aflatoxins are responsible for inhibiting the complement pathways. Chen *et al*. (2014) ^50^ noted in their study that the classical and alternative complement pathway of Pekin Ducklings was impaired when fed with 0.11 to 0.21mg/kg of aflatoxin B _1_. Valtchev *et al*. (2015) ^51^ also noted that low doses of AFB _1_ for a shorter period had immune stimulatory effects on Moulard Ducks, whereas high dosage for extended period repressed the alternative pathway of complement system. At different dosages, cattle and poultry fed with AFB _1_ had lower complement activity ^51, 52^. In general, the activity of the complement system is suppressed by AFB _1_.

### Effects on the adaptive immune system

The adaptive immune system is a type of immunity responsible for fighting foreign materials that have overwhelmed the innate immune system ^53^. The adaptive immune system is a system composed of T lymphocytes, B lymphocytes and antibodies ^54^. The suppression of adaptive immunity after consumption of aflatoxin B1 is well documented in the literature and this implies an increased susceptibility to pathogenic infection, as well as compromised protection even after immunization ^55^. A study by Tomková *et al*. (2002) demonstrated a remarkable reduction in the population of CD3 T cells in the mucosal membranes of the intestines of mice orally treated with aflatoxin B1. The decrease in the number of CD3 T cells implies a decrease in the host’s resistance to infectious pathogens. Similar effects have been documented in poultry through epidemiological studies associating AFB _1_ ingestion to insufficient protection provided by immunization against Newcastle disease ^51^. A similar study by Cysewski *et al.* (1978) ^56^ showed a failed protection of pigs fed with aflatoxin B _1_ against *Erysipelothrix rhusiopathiae* infection after vaccination.

Studies on AFB _1_ effects on T cells and expression of cytokines have been reported on animal models. Several studies have noted that exposure of AFB _1_ in animal models has a remarkable immunosuppressive effect as well as impact on humoral and cellular responses ^5, 57, 58^. Jiang *et al*. (2015a) ^59^ investigated the impact of aflatoxin B1 on the population of T-cell subclasses and the expression of cytokines in the small intestines of broilers. The population of T cells in the small intestines significantly decreased when compared to control group. Similarly, the expression of cytokines declined as compared to the control group.

The effect of AFB _1_ on antibodies has been studied extensively ^60, 61^. The study by Meissonnier *et al*. (2008) ^62^ showed that AFB _1_ exposure does not elicit a significant impact on the levels of IgA, IgG and IgM antibodies in the serum of pigs. Jiang *et al*. (2015b) ^63^ noted that Cobb broilers fed with AFB _1_ showed a significant reduction in the population of IgA (+) cells in the small intestine, duodenum and jejunum as compared to the untreated group. The mRNA expression levels of IgA, IgG and IgM in the lining of small intestines were significantly low in Cobb broilers fed with AFB _1_. However, Benkerroum (2020) ^64^ noted that feeding the rats with 0.1 or 1mg AFB _1_/kg b.w led to no change in anti-ovalbumin IgE and IgG antibody production in mesenteric lymph nodes even though it has profound impact on the proliferation of B and/or T cells. Similar findings have been documented by other researchers as well, especially a significant reduction in the levels of antibody after ingestion of AFB _1_, although it is worth noting that the effect was not similar across all animal species used.

## Effects of AFB _1_ on immune organs

The impact of AFB _1_ has been documented in a wide range of animal models. Peng *et al*. (2015) ^65^ conducted a study to investigate pathological changes in chicken immune organs upon ingesting AFB _1_ and AFB _2_. Histopathological examination showed that chickens fed with feeds contaminated with aflatoxins implicated nuclear debris in the thymus, bursa of Fabricius (BF) and spleen. A congestion of red pulp in the spleen was also observed, but not in the control group. The ultrastructure of the immune organs revealed abnormalities in the lymphocytes and reticulocytes of the thymus, BF, and spleen. It has also been demonstrated that ingestion of AFB _1_ has an effect on the weight of the immune organs. Guo *et al*. (2012) ^66^ did a study to investigate the impacts of aflatoxin B1 on the immune organs of ducklings. In a group treated with 0.1 mg/kg b.w., BF, thymus indexes, and body weights were significantly lower in comparison to the untreated group. Long *et al*. (2016) ^67^ in their study found out that AFB _1_ induces a reduction in body weight in mice, leading to a wasting away of immune organs. However, the ingestion of aflatoxin B _1_-contaminated maize feed implicated greater relative weight of spleen and BF (He *et al*. (2013) ^68^. Ingesting AFB _1_-contaminated maize significantly affects the body weight, immune system, and relative immune organ weight in ducks ^61, 68, 69^. It is therefore worth noting that the effect of AFB _1_ on the production traits and lower body weight of farm animals is a consequence of the worse utilization of feed proteins.

## Bioactive compounds and their mechanism of action

Plants possess a wide range of phytochemicals and most of them have their mechanism of action well elucidated. Some of the mechanism of actions that have been demonstrated include antimutagenic, antimicrobial, antioxidants, anticarcinogenic and anti-aflatoxic ^70– 72^. Wu *et al*., (2017) ^73^ demonstrated that some plant antioxidants, through their scavenging of free radicals, are capable of protecting the integrity of the cell membrane and macromolecules such as DNA. Plant extracts have also been demonstrated to act through the induction of xenobiotic detoxification and biotransformation pathways ^74^. Plant secondary metabolites such as polyphenols are able to inhibit the enzymes responsible for the activation of Phase I carcinogens ^75^. They have also been demonstrated to induce enzymes that facilitate Phase II detoxification processes ^76, 77^. The interference of enzymes in the two phases would affect metabolism of AFB _1_ to active metabolites. Certain compounds such as fungal probiotics and ferments have been used as additives in food and animal feed to prevent the growth of mold and contamination by harmful substances called mycotoxins ^78^. The use of these compounds have shown promise in controlling the risk of mutations and cancer caused by aflatoxins ^79^.

## Natural products: binders and metabolic modulators against Aflatoxin B _1_ immunotoxicity

Using natural products for medicinal purposes has been a longstanding practice. There is increasing interest in substances that can help reduce the impact of aflatoxin B _1_ on the immune system. Some studies have shown promising results in using these products to mitigate immunotoxic effects. For instance, a study conducted by Long *et al*. (2016) ^67^ discovered that grape seed proanthocyanidin extract (GSPE) can alleviate AFB _1_ induced immunotoxicity in mice. The study found that mice exposed to AFB _1_ experienced reduced organ weight and increased mortality compared to a control group. However, when administered with GSPE, the immune organ atrophy caused by AFB _1_ was ameliorated. Gao *et al.* (2021) ^80^ in their study demonstrated that morin is capable of protecting the liver and the kidneys of chicks against damage caused by AFB _1_.

Research has also been done to demonstrate the effects of binders such as bentonite clayin alleviating AFB _1_-induced immunotoxicity. The study by Bhatti *et al*. (2017) ^81^ found that the administration of bentonite clay alongside feeds significantly reduced AFB _1_-induced immunotoxicity in broilers. It was also observed that partial protection occurs at high AFB _1_ doses of up to 0.6mg/kg body weight. Other binders such as calcium montmorillonite clay has also been studied and found to reduce aflatoxin B1 biomarkers in rats. This implies immunoprotective effects against AFB _1_ (Mitchell *et al*. 2014) ^82^. In a study by Afriyie-Gyawu *et al*. (2008) ^83^ involving human subjects, a natural clay binder of the smectite group was used in the management of aflatoxicosis. The results indicated that smectites clay is effective in protecting humans against aflatoxin-induced toxicities. Lai *et al*. (2021) ^84^ and Zhao *et al*. (2021) ^85^ conclusively noted that commercial mycotoxins-binder, XL, at a dose of 2g per kg body weight and other mycotoxin binders such as TOXO HP can ameliorate the immunotoxic effects of AFB _1_ on broilers. Saleemi *et al.* (2020) ^80^ reported that local mycotoxin binder (LMB) can be used to combat the toxicopathological effects caused by AFB _1_ in poultry. Their study demonstrated that the effectiveness of LMB in combating the undesirable consequences is dependent on LMB dose. Selenium has also been shown to protect against the toxic effects of aflatoxins (Chen *et al*. 2014; Liang *et al*. 2015; Singh *et al*. 2006) ^86– 88^. Chand *et al*. (2011) ^89^ found out in their study that milk thistle at a dose of 10g/kg of feed can effectually be used as an immune-stimulant and in growth promotion in the presence of immunosuppressant AFB _1_ in the feedstuff.

According to Singh *et al.* (2019), crude extracts of *Premna integrifolia* inhibits aflatoxin B _1_ toxicity in mice. Aflatoxin B _1_ induces toxicity in the liver which can be reversed by oral administration of *Premna integrifolia*. Singh *et al.* (2019) also demonstrated that crude extracts of *Premna integrifolia* restore hematological and serum parameters while also reducing the oxidative stress and hepatotoxicity caused by AFB _1_^90^. Similarly, crude extracts of *Annona senegalensis* inhibit genotoxic effects resulting from AFB _1_ in *in vitro* assays and this implies its role in cancer prevention ^91^. In addition, *in vitro* studies involving crude extracts of *Monanthotaxis caffra* were shown to have inhibitory effect against AFB _1_ genotoxicity ^91, 92^. Pauletto *et al.* (2021) ^93^ demonstrated the ameriolative effects of resveratrol on AFB _1_-induced toxicities. The findings of their study add to the understanding of important cellular processes associated with antioxidant-mediated defense against AFB _1_ toxic effects. Consumption of food additives that modulate immune system have also been shown to be a promising approach in fight against aflatoxicosis ^61, 69^. In a study by Kipkoech *et al*. (2023) ^69^, crude extract of *Spirulina plantesis* could be used as an immune booster thus countering AFB _1_ induced immune dysfunction and inflammation.

## Future direction and conclusions

Aflatoxicosis is a worldwide problem. Research on the immunomodulation in AFB _1_ toxicity has not been extensively done and there is a need to understand the mechanism of AFB _1_-induced immunotoxicity. Aflatoxicosis is a serious, life-threatening problem caused by AFB _1_ and other mycotoxins in the same family. It is one of the main causes of global deaths in people aged 20 years or older and about 25,200–155,000 annual hepatocellular carcinomas are directly linked to aflatoxin exposure ^94^. There is an urgent need to develop safer feed additives that can be used in aflatoxicosis management. The use of novel approaches such as of bentonite clay, smectites clay, GSPE and other approaches highlighted in this paper would help solve this public health problem. Several other feed additives have shown promise, but still require further research for their use as immunomodulators.

A comprehensive literature review has pointed out that aflatoxin B _1_ is the most potent mycotoxin. The health implication of ingesting this toxin is a serious public health concern. The existing approaches that can be used to manage aflatoxicosis are not very effective, which calls for more research to understand the exact mechanism of AFB _1_ immunotoxity and develop effective control measures. Aflatoxicosis not only affects humans but also domestic animals. The consumption of animal products from animals that have consumed AFB _1_-contaminated feeds worsens the situation. As advancements in research are ongoing to develop effective interventions, the ultimate approach that can be used to reduce aflatoxin exposure is through proper management of cereals from harvesting to storage. Studies have noted that proper drying and storage of cereals prevents *Aspergillus flavus* growth and toxin production.

## Data Availability

No data are associated with this article.
