# Supplementary material for: Immunomodulatory effects of aflatoxin B1 (AFB1) and the use of natural products to ameliorate its immunotoxic effects: A review
Source: Open Res Afr. 2025 Feb 7;6:22. Originally published 2023 Dec 14. [Version 2] doi: 10.12688/openresafrica.14406.2 (PMC11885904; doi:10.12688/openresafrica.14406.2)
Supplement: Peer review history

# Reviewer report: Reviewer response for version 2

**DOI:** 10.21956/openresafrica.16864.r32135

**Published:** 2025-03-06

**Authors:** Samir Abbès

**Affiliations:** 1 Higher Institute of Biotechnology of Monastir, University of Monastir, Monastir, Tunisia

The paper entitled Immunomodulatory effects of aflatoxin B1 (AFB1) and the use of natural products to ameliorate its immunotoxic effects: A review, is well written and very informative but the following suggestions should be addressed.

- In the abstract section, Many other products and extracts from plants are used against Aflatoxine’s immunotoxicity like Radish,….Jebali *et al* (2015 ^1^), Abbès *et al *(2016 ^2^), Ben Salah-Abbès *et al* (2015 ^3^). (please add information for the readers, both in abstract and introduction section, and plant extract section).
- Keywords section: The review discuss also the amelioration of the AFB1 immunotoxicities, its preferable to add in the keywords ….prevention/ameliorate… 
- Add a section for AFB1 regulation and risk assessment and discuss it with the used dose causing immunotoxicities.
- Add table with AFB1 doses and immune target organs to give more information to the readers.

Is the review written in accessible language?

Yes

Are all factual statements correct and adequately supported by citations?

Yes

Are the conclusions drawn appropriate in the context of the current research literature?

Yes

Is the topic of the review discussed comprehensively in the context of the current literature?

Yes

Reviewer Expertise:

Immunotoxicity of mycotoxins

I confirm that I have read this submission and believe that I have an appropriate level of expertise to confirm that it is of an acceptable scientific standard, however I have significant reservations, as outlined above.

## References

[1] : Ability of Lactobacillus plantarum MON03 to mitigate aflatoxins (B1 and M1) immunotoxicities in mice. *J Immunotoxicol* .2015;12(3) : 10.3109/1547691X.2014.973622 290-9 10.3109/1547691X.2014.973622 25441623

[2] : Interaction of aflatoxin B1 and fumonisin B1 in mice causes immunotoxicity and oxidative stress: Possible protective role using lactic acid bacteria. *J Immunotoxicol* .2016;13(1) : 10.3109/1547691X.2014.997905 46-54 10.3109/1547691X.2014.997905 25585958

[3] : Immuno-physiological alterations from AFB1 in rats counteracted by treatments with Lactobacillus paracasei BEJ01 and montmorillonite clay mixture. *J Immunotoxicol* .2016;13(5) : 10.3109/1547691X.2016.1145157 628-37 10.3109/1547691X.2016.1145157 27294391

# Reviewer report: Reviewer response for version 1

**DOI:** 10.21956/openresafrica.15564.r30532

**Published:** 2024-11-22

**Authors:** Miklós Mézes

**Affiliations:** 1 Mycotoxins in the Food Chain Research Group, Hungarian University of Agriculture and Life Sciences, Kaposvár, Hungary

**Comments:**

- NovaSil Clay is a trademark. Please avoid adding a trademark in a scientific paper. Please specify the clay.
- Please use the term mold instead of fungus.
- The statement’ hepatocarcinogen in animal models’ is partly true because the IARC category of AFB1 is Group 1 human carcinogen.
- Please mention that the hydroxylated metabolite AFM1 is excreted through the milk, and this way would be important in aflatoxicosis in young mammals and infants.
- Figure 1: Please modify that AFB1 is produced by other Aspergillus species, e.g., *A. parasiticus* and others. For this reason, it would be better to use the term, e.g *., A. flavus*
- Please mention in the manuscript that the effect of AFB1 on the production traits and lower body weight of farm animals is a consequence of the worse utilization of feed proteins.
- Restructuring the subchapter “Use of natural products to alleviate immunotoxic effects of aflatoxin B1” requires. For instance, medicinal herbs, clays, and others (e.g., selenium). Its present form is a mix of products with different modes of action. This subchapter must mention which products are binders and which act within the metabolism in different organs.

Is the review written in accessible language?

Yes

Are all factual statements correct and adequately supported by citations?

Partly

Are the conclusions drawn appropriate in the context of the current research literature?

Yes

Is the topic of the review discussed comprehensively in the context of the current literature?

Yes

Reviewer Expertise:

Mycotoxin research with particular interest of oxidative stress

I confirm that I have read this submission and believe that I have an appropriate level of expertise to confirm that it is of an acceptable scientific standard.

# Response

**Published:** 2025-01-07

**Authors:** Gilbert Kipkoech

**Affiliations:** Centre for Traditional Medicine and Drug Research, Kenya Medical Research Institute, Kenya

Thank you for your comments. I have addressed them accordingly.

# Reviewer report: Reviewer response for version 1

**DOI:** 10.21956/openresafrica.15564.r30705

**Published:** 2024-02-24

**Authors:** Francis Padi Lamptey

**Affiliations:** 1 Department of Food Science and Postharvest Technology, Cape Coast Technical University (Ringgold ID: 233753), Cape Coast, Central, Ghana

This review summarized the interaction between AFB _1_ and the host immune system. It highlights other potential mechanisms involved in AFB1-induced immunosuppression. It discusses findings on natural products that have been found capable of inhibiting AFB1-induced immunosuppression in some animal models. This is an interesting and good work done. However, I think the authors should pay attention to the following:

 1. Aflatoxin B1 or any type of Aflatoxin should be written as subscript; say, B _1_, B _2_, G _1_, G _2_, etc.

 2. Remove the statement recent findings or explain why you are saying recent findings when you clearly stated in your methodology that age was not considered when choosing articles to write this paper.

 3. Reference 95 should be taken into the work. It is not very appropriate to include a reference in the conclusion.

 4. The sub-heading methodology has systematic review. This should be taken out since this is not a systematic review. For more information on systematic review, read on, PRISMA in systematic review.

 5. Saleemi et al. (2020) does not have a superscript to show that it is a reference. Kindly do it well.

Is the review written in accessible language?

Yes

Are all factual statements correct and adequately supported by citations?

Yes

Are the conclusions drawn appropriate in the context of the current research literature?

Yes

Is the topic of the review discussed comprehensively in the context of the current literature?

Yes

Reviewer Expertise:

Mycotoxins, Non-Destructive Measurement, Food Fraud, Food Quality and Food Safety.

I confirm that I have read this submission and believe that I have an appropriate level of expertise to confirm that it is of an acceptable scientific standard, however I have significant reservations, as outlined above.

# Response

**Published:** 2025-01-07

**Authors:** Gilbert Kipkoech

**Affiliations:** Centre for Traditional Medicine and Drug Research, Kenya Medical Research Institute, Kenya

Than you for your comments.
